# 4-Hy­droxy-*N*′-[1-(2-hy­droxy­phen­yl)ethyl­idene]benzohydrazide

**DOI:** 10.1107/S1600536810019914

**Published:** 2010-05-29

**Authors:** Xiao-Hui Ji, Jiu-Fu Lu

**Affiliations:** a School of Chemistry and Environmental Science, Shaanxi University of Technology, Hanzhong 723000, People’s Republic of China

## Abstract

In the title compound, C_15_H_14_N_2_O_3_,  there is an intra­molecular O—H⋯N hydrogen bond and the dihedral angle between the two aromatic rings is 13.9 (3)°. In the crystal structure, mol­ecules are stabilized by inter­molecular O—H⋯O and N—H⋯O hydrogen bonds.

## Related literature

For related structures, see: Lu *et al.* (2008*a* ▶,*b* ▶,*c* ▶); Xiao & Wei (2009 ▶); He (2008 ▶); Shi *et al.* (2007 ▶). For bond-length data, see: Allen *et al.* (1987 ▶).
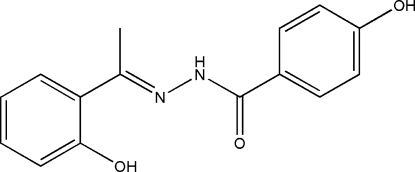


## Experimental

### 

#### Crystal data

C_15_H_14_N_2_O_3_
*M* *_r_* = 270.28
Monoclinic, 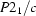

*a* = 4.926 (2) Å
*b* = 31.06 (2) Å
*c* = 8.473 (3) Å
β = 93.852 (3)°
*V* = 1293.5 (11) Å^3^
*Z* = 4
Mo *K*α radiation
μ = 0.10 mm^−1^
*T* = 298 K
0.21 × 0.20 × 0.17 mm

#### Data collection

Bruker APEXII CCD area-detector diffractometer
Absorption correction: multi-scan (*SADABS*; Sheldrick, 2004 ▶) *T*_min_ = 0.980, *T*_max_ = 0.984
8965 measured reflections
2770 independent reflections
1569 reflections with *I* > 2σ(*I*)
*R*_int_ = 0.064

#### Refinement

*R*[*F* ^2^ > 2σ(*F* ^2^)] = 0.069
*wR*(*F* ^2^) = 0.172
*S* = 1.05
2770 reflections
188 parameters
1 restraint
H atoms treated by a mixture of independent and constrained refinement
Δρ_max_ = 0.26 e Å^−3^
Δρ_min_ = −0.21 e Å^−3^

### 

Data collection: *APEX2* (Bruker, 2004 ▶); cell refinement: *SAINT* (Bruker, 2004 ▶); data reduction: *SAINT*; program(s) used to solve structure: *SHELXS97* (Sheldrick, 2008 ▶); program(s) used to refine structure: *SHELXL97* (Sheldrick, 2008 ▶); molecular graphics: *SHELXTL* (Sheldrick, 2008 ▶); software used to prepare material for publication: *SHELXTL*.

## Supplementary Material

Crystal structure: contains datablocks global, I. DOI: 10.1107/S1600536810019914/su2181sup1.cif

Structure factors: contains datablocks I. DOI: 10.1107/S1600536810019914/su2181Isup2.hkl

Additional supplementary materials:  crystallographic information; 3D view; checkCIF report

## Figures and Tables

**Table 1:** Hydrogen-bond geometry (Å, °)

*D*—H⋯*A*	*D*—H	H⋯*A*	*D*⋯*A*	*D*—H⋯*A*
O1—H1⋯N1	0.82	1.78	2.499 (3)	146
O3—H3⋯O1^i^	0.82	1.97	2.786 (3)	179
N2—H2⋯O2^ii^	0.90 (1)	2.09 (2)	2.961 (4)	163 (3)

Symmetry codes: (i) 
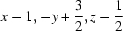
; (ii) 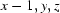.

**Fig. 1.**
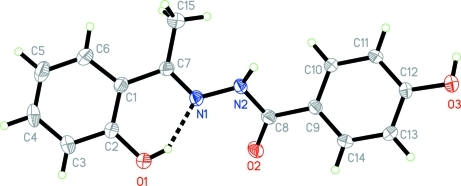
The molecular structure of the title compound, showing 30% probability displacement ellipsoids and the atom-numbering scheme. The dashed line indicates the intramolecular O-H···N hydrogen bond (see Table 1 for details.

**Fig. 2.**
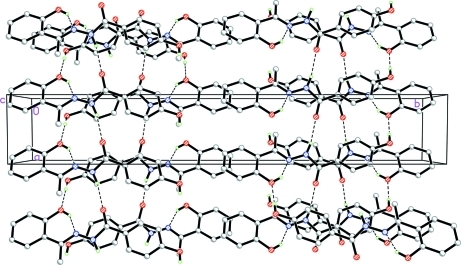
The crystal packing of the title compound, viewed along the c axis. H-atoms not involved in hydrogen bonding (dashed lines) have been omitted for clarity.

## supplementary crystallographic information

### Comment

As part of our investigation of the crystal structures of Schiff bases derived from the condensation of aldehydes or ketones with benzohydrazides (Lu *et al.*, 2008*a*,b,c), we report herein on the crystal structure of the new title Schiff base compound.

In the title compound, illustrated in Fig. 1, the bond lengths have normal values (Allen *et al.*, 1987), and are comparable to those observed in similar compounds (Xiao & Wei, 2009; He, 2008; Shi *et al.*, 2007). The dihedral angle between the two aromatic rings is 13.9 (3)°, indicating that the Schiff base molecule is slightly twisted. An intramolecular O—H···N hydrogen bond is observed (Table 1).

In the crystal structure, the molecules are stabilized by intermolecular O—H···O and N—H···O hydrogen bonds (Table 1 and Fig. 2).

### Experimental

The title compound was prepared by the Schiff base condensation of 1-(2-hydroxyphenyl)ethanone (0.1 mol, 13.6 g) and 4-hydroxybenzohydrazide (0.1 mmol, 15.2 g) in 95% ethanol (70 ml). The excess ethanol was removed by distillation. The colourless solid obtained was filtered and washed with ethanol. Single crystals, suitable for X-ray diffraction analysis, were obatined by slow evaporation of a 95% ethanol solution at rt.

### Refinement

The N2 H-atom (H2) was located in a difference Fourier map and refined with a distance restraint: N-H = 0.90 (1) Å. The other H-atoms were positioned geometrically (C—H = 0.93–0.96 Å and O—H = 0.82 Å) and refined using a riding model, with *U*_iso_(H) = 1.2*U*_eq_(C) and 1.5*U*_eq_(C_methyl_ and O). A rotating group model was used for the methyl and hydroxyl groups.

### Crystal data

**Table:**

C_15_H_14_N_2_O_3_	*F*(000) = 568
*M**_r_* = 270.28	*D*_x_ = 1.388 Mg m^−^^3^
Monoclinic, *P*2_1_/*c*	Mo *K*α radiation, λ = 0.71073 Å
Hall symbol: -P 2ybc	Cell parameters from 957 reflections
*a* = 4.926 (2) Å	θ = 2.5–24.5°
*b* = 31.06 (2) Å	µ = 0.10 mm^−^^1^
*c* = 8.473 (3) Å	*T* = 298 K
β = 93.852 (3)°	Block, colourless
*V* = 1293.5 (11) Å^3^	0.21 × 0.20 × 0.17 mm
*Z* = 4	

### Data collection

**Table:**

Bruker APEXII CCD area-detector diffractometer	2770 independent reflections
Radiation source: fine-focus sealed tube	1569 reflections with *I* > 2σ(*I*)
graphite	*R*_int_ = 0.064
ω scans	θ_max_ = 27.0°, θ_min_ = 2.5°
Absorption correction: multi-scan (*SADABS*; Sheldrick, 2004)	*h* = −6→6
*T*_min_ = 0.980, *T*_max_ = 0.984	*k* = −39→34
8965 measured reflections	*l* = −10→10

### Refinement

**Table:**

Refinement on *F*^2^	Secondary atom site location: difference Fourier map
Least-squares matrix: full	Hydrogen site location: inferred from neighbouring sites
*R*[*F*^2^ > 2σ(*F*^2^)] = 0.069	H atoms treated by a mixture of independent and constrained refinement
*wR*(*F*^2^) = 0.172	*w* = 1/[σ^2^(*F*_o_^2^) + (0.0649*P*)^2^ + 0.4306*P*] where *P* = (*F*_o_^2^ + 2*F*_c_^2^)/3
*S* = 1.05	(Δ/σ)_max_ < 0.001
2770 reflections	Δρ_max_ = 0.26 e Å^−^^3^
188 parameters	Δρ_min_ = −0.21 e Å^−^^3^
1 restraint	Extinction correction: *SHELXL97* (Sheldrick, 2008), Fc^*^=kFc[1+0.001xFc^2^λ^3^/sin(2θ)]^-1/4^
Primary atom site location: structure-invariant direct methods	Extinction coefficient: 0.0057 (18)

### Special details

**Table:**

Geometry. All e.s.d.'s (except the e.s.d. in the dihedral angle between two l.s. planes) are estimated using the full covariance matrix. The cell e.s.d.'s are taken into account individually in the estimation of e.s.d.'s in distances, angles and torsion angles; correlations between e.s.d.'s in cell parameters are only used when they are defined by crystal symmetry. An approximate (isotropic) treatment of cell e.s.d.'s is used for estimating e.s.d.'s involving l.s. planes.
Refinement. Refinement of *F*^2^ against ALL reflections. The weighted *R*-factor *wR* and goodness of fit *S* are based on *F*^2^, conventional *R*-factors *R* are based on *F*, with *F* set to zero for negative *F*^2^. The threshold expression of *F*^2^ > σ(*F*^2^) is used only for calculating *R*-factors(gt) *etc*. and is not relevant to the choice of reflections for refinement. *R*-factors based on *F*^2^ are statistically about twice as large as those based on *F*, and *R*- factors based on ALL data will be even larger.

### Fractional atomic coordinates and isotropic or equivalent isotropic displacement parameters (Å^2^)

**Table:**

	*x*	*y*	*z*	*U*_iso_*/*U*_eq_	
N1	0.9548 (5)	0.64659 (7)	0.6666 (3)	0.0384 (6)	
N2	0.8786 (5)	0.68800 (7)	0.6186 (3)	0.0389 (6)	
O1	1.2807 (5)	0.60332 (7)	0.8408 (3)	0.0581 (7)	
H1	1.2112	0.6252	0.8021	0.087*	
O2	1.3097 (4)	0.71011 (6)	0.6779 (3)	0.0510 (6)	
O3	0.7088 (5)	0.89007 (6)	0.5716 (3)	0.0593 (7)	
H3	0.5810	0.8920	0.5048	0.089*	
C1	0.9088 (6)	0.57209 (9)	0.6782 (3)	0.0377 (7)	
C2	1.1338 (6)	0.56842 (9)	0.7889 (3)	0.0413 (8)	
C3	1.2167 (7)	0.52855 (10)	0.8469 (4)	0.0530 (9)	
H3A	1.3668	0.5266	0.9192	0.064*	
C4	1.0804 (8)	0.49197 (10)	0.7992 (4)	0.0603 (10)	
H4	1.1375	0.4653	0.8389	0.072*	
C5	0.8616 (8)	0.49468 (11)	0.6938 (5)	0.0644 (11)	
H5	0.7681	0.4698	0.6618	0.077*	
C6	0.7776 (7)	0.53373 (10)	0.6342 (4)	0.0544 (9)	
H6	0.6274	0.5348	0.5617	0.065*	
C7	0.8165 (6)	0.61399 (9)	0.6136 (3)	0.0366 (7)	
C8	1.0701 (6)	0.71922 (9)	0.6405 (3)	0.0374 (7)	
C9	0.9732 (6)	0.76376 (9)	0.6167 (3)	0.0346 (7)	
C10	0.7529 (6)	0.77407 (9)	0.5133 (3)	0.0394 (7)	
H10	0.6639	0.7523	0.4547	0.047*	
C11	0.6634 (6)	0.81585 (9)	0.4958 (3)	0.0411 (8)	
H11	0.5158	0.8222	0.4254	0.049*	
C12	0.7927 (6)	0.84846 (9)	0.5827 (3)	0.0392 (7)	
C13	1.0155 (6)	0.83870 (10)	0.6841 (4)	0.0477 (8)	
H13	1.1056	0.8605	0.7415	0.057*	
C14	1.1051 (6)	0.79695 (10)	0.7005 (4)	0.0446 (8)	
H14	1.2560	0.7908	0.7687	0.054*	
C15	0.5813 (7)	0.61695 (11)	0.4922 (4)	0.0541 (9)	
H15A	0.4183	0.6075	0.5378	0.081*	
H15B	0.6154	0.5990	0.4035	0.081*	
H15C	0.5595	0.6463	0.4575	0.081*	
H2	0.710 (3)	0.6991 (11)	0.622 (4)	0.080*	

### Atomic displacement parameters (Å^2^)

**Table:**

	*U*^11^	*U*^22^	*U*^33^	*U*^12^	*U*^13^	*U*^23^
N1	0.0324 (14)	0.0301 (14)	0.0522 (15)	0.0026 (11)	0.0000 (11)	0.0024 (11)
N2	0.0325 (14)	0.0302 (14)	0.0531 (15)	0.0022 (11)	−0.0030 (12)	0.0062 (11)
O1	0.0596 (15)	0.0364 (13)	0.0744 (16)	−0.0035 (11)	−0.0252 (12)	0.0046 (11)
O2	0.0294 (12)	0.0443 (13)	0.0784 (16)	0.0045 (10)	−0.0033 (11)	0.0088 (11)
O3	0.0686 (18)	0.0336 (13)	0.0721 (17)	0.0086 (11)	−0.0224 (13)	−0.0024 (10)
C1	0.0364 (17)	0.0327 (16)	0.0439 (16)	−0.0017 (13)	0.0030 (13)	−0.0025 (13)
C2	0.0458 (19)	0.0313 (17)	0.0464 (17)	−0.0017 (14)	0.0001 (14)	−0.0005 (13)
C3	0.062 (2)	0.0367 (19)	0.060 (2)	0.0050 (16)	−0.0040 (17)	0.0084 (15)
C4	0.077 (3)	0.0292 (19)	0.076 (2)	0.0094 (17)	0.013 (2)	0.0120 (16)
C5	0.072 (3)	0.0326 (19)	0.088 (3)	−0.0091 (18)	0.000 (2)	−0.0022 (17)
C6	0.055 (2)	0.041 (2)	0.066 (2)	−0.0074 (16)	−0.0070 (17)	−0.0049 (16)
C7	0.0340 (17)	0.0351 (16)	0.0405 (16)	0.0006 (13)	−0.0001 (13)	−0.0023 (12)
C8	0.0338 (17)	0.0386 (17)	0.0397 (16)	0.0010 (14)	0.0017 (13)	0.0043 (13)
C9	0.0314 (16)	0.0334 (16)	0.0388 (15)	0.0013 (13)	0.0017 (12)	0.0036 (12)
C10	0.0415 (18)	0.0313 (16)	0.0438 (17)	−0.0016 (13)	−0.0080 (13)	−0.0003 (13)
C11	0.0426 (18)	0.0367 (17)	0.0419 (17)	0.0008 (14)	−0.0125 (14)	0.0048 (13)
C12	0.0450 (18)	0.0277 (16)	0.0445 (17)	0.0026 (13)	−0.0013 (14)	0.0032 (13)
C13	0.046 (2)	0.0353 (18)	0.0588 (19)	−0.0043 (14)	−0.0146 (16)	−0.0076 (15)
C14	0.0380 (18)	0.0428 (18)	0.0509 (19)	0.0019 (15)	−0.0127 (14)	0.0019 (14)
C15	0.049 (2)	0.053 (2)	0.058 (2)	−0.0024 (17)	−0.0111 (16)	0.0030 (16)

### Geometric parameters (Å, °)

**Table:**

N1—C7	1.284 (3)	C5—H5	0.9300
N1—N2	1.393 (3)	C6—H6	0.9300
N2—C8	1.357 (4)	C7—C15	1.500 (4)
N2—H2	0.900 (10)	C8—C9	1.473 (4)
O1—C2	1.360 (3)	C9—C10	1.385 (4)
O1—H1	0.8200	C9—C14	1.388 (4)
O2—C8	1.234 (3)	C10—C11	1.375 (4)
O3—C12	1.358 (3)	C10—H10	0.9300
O3—H3	0.8200	C11—C12	1.382 (4)
C1—C6	1.394 (4)	C11—H11	0.9300
C1—C2	1.407 (4)	C12—C13	1.381 (4)
C1—C7	1.472 (4)	C13—C14	1.374 (4)
C2—C3	1.384 (4)	C13—H13	0.9300
C3—C4	1.367 (4)	C14—H14	0.9300
C3—H3A	0.9300	C15—H15A	0.9600
C4—C5	1.356 (5)	C15—H15B	0.9600
C4—H4	0.9300	C15—H15C	0.9600
C5—C6	1.367 (5)		
			
C7—N1—N2	120.0 (2)	O2—C8—N2	121.0 (3)
C8—N2—N1	116.7 (2)	O2—C8—C9	123.1 (3)
C8—N2—H2	111 (2)	N2—C8—C9	115.9 (2)
N1—N2—H2	125 (2)	C10—C9—C14	118.1 (3)
C2—O1—H1	109.5	C10—C9—C8	122.5 (3)
C12—O3—H3	109.5	C14—C9—C8	119.4 (2)
C6—C1—C2	116.1 (3)	C11—C10—C9	121.2 (3)
C6—C1—C7	122.0 (3)	C11—C10—H10	119.4
C2—C1—C7	121.9 (2)	C9—C10—H10	119.4
O1—C2—C3	117.4 (3)	C10—C11—C12	120.1 (3)
O1—C2—C1	122.0 (2)	C10—C11—H11	120.0
C3—C2—C1	120.6 (3)	C12—C11—H11	120.0
C4—C3—C2	120.7 (3)	O3—C12—C13	118.5 (3)
C4—C3—H3A	119.6	O3—C12—C11	122.2 (3)
C2—C3—H3A	119.6	C13—C12—C11	119.3 (3)
C5—C4—C3	119.8 (3)	C14—C13—C12	120.4 (3)
C5—C4—H4	120.1	C14—C13—H13	119.8
C3—C4—H4	120.1	C12—C13—H13	119.8
C4—C5—C6	120.4 (3)	C13—C14—C9	120.9 (3)
C4—C5—H5	119.8	C13—C14—H14	119.6
C6—C5—H5	119.8	C9—C14—H14	119.6
C5—C6—C1	122.4 (3)	C7—C15—H15A	109.5
C5—C6—H6	118.8	C7—C15—H15B	109.5
C1—C6—H6	118.8	H15A—C15—H15B	109.5
N1—C7—C1	115.0 (2)	C7—C15—H15C	109.5
N1—C7—C15	124.0 (3)	H15A—C15—H15C	109.5
C1—C7—C15	121.0 (3)	H15B—C15—H15C	109.5

### Hydrogen-bond geometry (Å, °)

**Table:**

*D*—H···*A*	*D*—H	H···*A*	*D*···*A*	*D*—H···*A*
O1—H1···N1	0.82	1.78	2.499 (3)	146
O3—H3···O1^i^	0.82	1.97	2.786 (3)	179
N2—H2···O2^ii^	0.90 (1)	2.09 (2)	2.961 (4)	163 (3)

Symmetry codes: (i) *x*−1, −*y*+3/2, *z*−1/2; (ii) *x*−1, *y*, *z*.
